# Fatigue Strength Improvement of Laser-Directed Energy Deposition 316L Stainless Steel with In Situ Ultrasonic Rolling by Preliminary Investigation

**DOI:** 10.3390/ma17153693

**Published:** 2024-07-26

**Authors:** Guan Liu, Yigui Su, Xuyu Pi, Defu Liu, Yongcheng Lin

**Affiliations:** School of Mechanical and Electrical Engineering, Central South University, Changsha 410083, China; 223712101@csu.edu.cn (Y.S.); 233711009@csu.edu.cn (X.P.)

**Keywords:** laser-directed energy deposition, ultrasonic rolling, 316L stainless steel, microstructure, fatigue behavior

## Abstract

In this study, to improve the fatigue strength of the LDED (laser-directed energy deposition) 316L stainless steel, an in situ ultrasonic rolling technology is developed to assist the laser-directed energy deposition process (LDED-UR). The microstructural characteristics and fatigue behavior are comprehensively discussed. The results show that the average size of pores of the LDED-UR alloy is about 10.2 μm, which is much smaller than that of the LDED alloy (34.1 μm). Meanwhile, the density of the LDED alloy is also enhanced from 98.26% to 99.27% via the in situ ultrasonic rolling. With the application of the in situ ultrasonic rolling, the grains are transformed into fully equiaxed grains, and their average grain size is greatly reduced from 84.56 μm to 26.93 μm. The fatigue limit of the LDED-UR alloy is increased by 29% from 210 MPa (LDED alloy) to 270 MPa, which can be ascribed to the decreased porosity and the fine grains. In particular, the crack initiation site of the LDED alloy is located at the surfaces, while it is nucleated from the sub-surface for the LDED-UR alloy. This is mainly attributed to the compression residual stress induced by the in situ ultrasonic rolling. This research offers a valuable understanding of the failure mechanisms in additively manufactured metals, guiding the development of effective strategies to improve their fatigue threshold under severe operating conditions.

## 1. Introduction

Due to its superior corrosion resistance and weld-ability, 316L stainless steel has been extensively employed in medical, marine, aerospace, and other industrial fields [1,2,3]. In such applications, components have complex geometries and are often customized [4,5,6]. Traditional manufacturing methods, involving casting, forging, machining, etc., are time-consuming [7,8]. These methods also incur high costs for custom products [9,10]. Laser-directed energy deposition (LDED), as an innovative near-net forming technology, has gained extensive attention in academia and industry, and it is a viable means of fabricating customized complex components [11,12,13,14,15,16,17].

Some researchers have focused on manufacturing 316L stainless steel by laser-directed energy deposition technology [18,19,20]. Ziętala et al. [21] employed laser-directed energy deposition technology to fabricate 316L stainless steel alloy, and found that a significant increase in Cr and Mo content and a decrease in Ni appear in the grain boundaries. This has a visible impact on the mechanical properties. DeNonno et al. [22] found that the microstructure of laser-deposited 316L stainless steel is strongly correlated with the composition and solidification conditions, and discovered that the accelerated solidification velocity transforms the solidification mode from primary δ-ferrite to primary austenite. Lai et al. [23] enhanced the corrosion resistance and mechanical properties of the laser-deposited 316L stainless steel by adding element W, and found that the precipitated WO_3_ was the main reason for the improvement of its properties. It was found that the components are typically susceptible to defects, such as pores, which result in weakened properties [24]. In particular, the fatigue strength, as a key evaluation for structural components, is known to be more sensitive to defects than the quasi-static properties [25]. For this reason, the additively manufactured components express low fatigue strength, while they may exhibit better tensile strength compared with the wrought parts [26].

Many studies have been conducted to enhance the fatigue strength of additively manufactured alloys [27,28,29]. Raikar et al. [30] employed a sulfur-based post-processing technique to reduce the surface roughness, and found that the tension–tension fatigue life increased by 340% from about 7000 cycles to 30,000 cycles. Laser shock peening is used by Singh et al. [31] to study the relationship between the defects and properties of the LDED alloy. The results show that proper depositions without major defects and cracks are limited to the surface. Meanwhile, the microhardness can be significantly improved by 49.81% after laser peening, while the fatigue strength is not enhanced for all samples. Barr et al. [32] found that the high-cycle fatigue strength is correlated to crack initiation at defects or locally softened regions for in situ tempering. Concentrated crack growth and premature failure can be prevented by employing uniform tempering. Blinn et al. [33] studied the effects of heat treatment on the fatigue behavior of additively manufactured AISI 316L stainless steel, and discovered that the fatigue strength is reduced due to the coarse grains. Although researchers have developed some strategies to enhance fatigue strength, new and improved methods are urgently needed for engineering applications.

For the alloys fabricated by traditional processes, the severe plastic deformation (SPD) process is an effective route in enhancing its fatigue property [34,35]. Hosseini et al. [36] employed equal channel angular pressing technology to induce the formation of compressive residual stresses, and found that the growth rate of fatigue cracks was suppressed, i.e., the fatigue life was promoted. Miková et al. [37] enhanced the tensile strength of X70 microalloyed steel after severe shot peening. Lei et al. [38] synthesized a gradient nanostructured surface layer with full austenitic phase by surface mechanical rolling treatment at 280 °C, and demonstrated a high fatigue strength and endurance due to the suppressed deformation-induced martensite transformation and refined grains. Could the plastic deformation method also enhance the fatigue property of the additively manufactured alloys?

Here, an innovative plastic deformation technology, i.e., ultrasonic rolling, is introduced to eliminate the pore defects, and then enhance the fatigue strength. To the best of the authors’ knowledge, very few studies have addressed this technique to synchronously assist the laser-directed energy deposition process. In this study, an in situ ultrasonic rolling assisted LDED device is developed to manufacture 316L stainless steel. Firstly, the developed LDED-UR device is introduced in detail. Meanwhile, the microstructure analysis methods and property analysis methods are also addressed. Secondly, the pores’ features and grain characterization are discussed for the LDED and LDED-UR alloys. Thirdly, the microhardness and fatigue behavior of the LDED and LDED-UR alloys are studied. Lastly, the improvement mechanisms of fatigue resistance for the LDED-UR alloy are deeply analyzed. A comprehensive understanding of the relationship between the microstructure and fatigue behavior provides technical guidance for improving the fatigue strength of the alloy produced by LDED.

## 2. Materials and Methods

### 2.1. Materials

**Figure 1:**
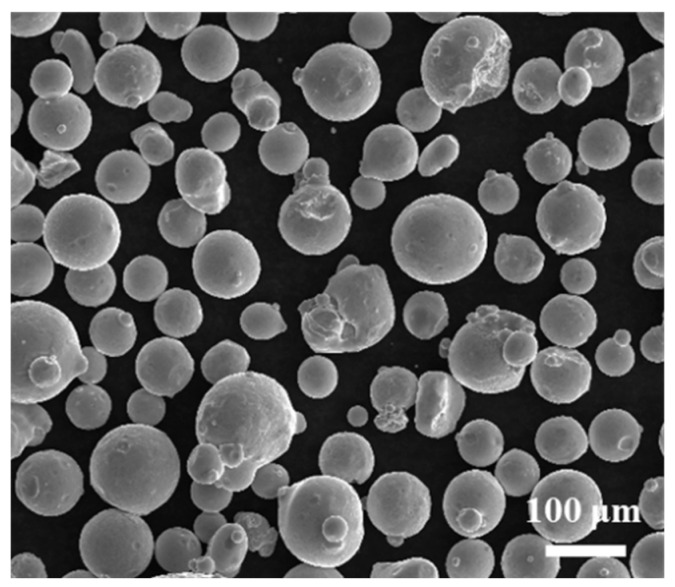
SEM images of the 316L powders used.

Experiments were conducted on 316L specimens manufactured by conventional methods, and the thickness of the plate was 16 mm. The plates are polished with sandpaper to remove the oxide film and then cleaned with deionized water. The 316L ‘stock’ materials (AVIC Maite, Beijing, China) with an average size of 70 μm are employed, and their morphologies are presented in Figure 1. The compositions for the employed 316L alloy are 0.024 C, 2.55 Mo, 17.03 Cr, 0.45 Mn,10.64 Ni, 0.11 Si, and balance Fe. Before testing, the powders are pre-dried for 12 h at 100 °C. During the LDED process, 99.99% Ar gas is used to prevent the melt pool from oxidation.

### 2.2. Manufacturing Methods

**Figure 2:**
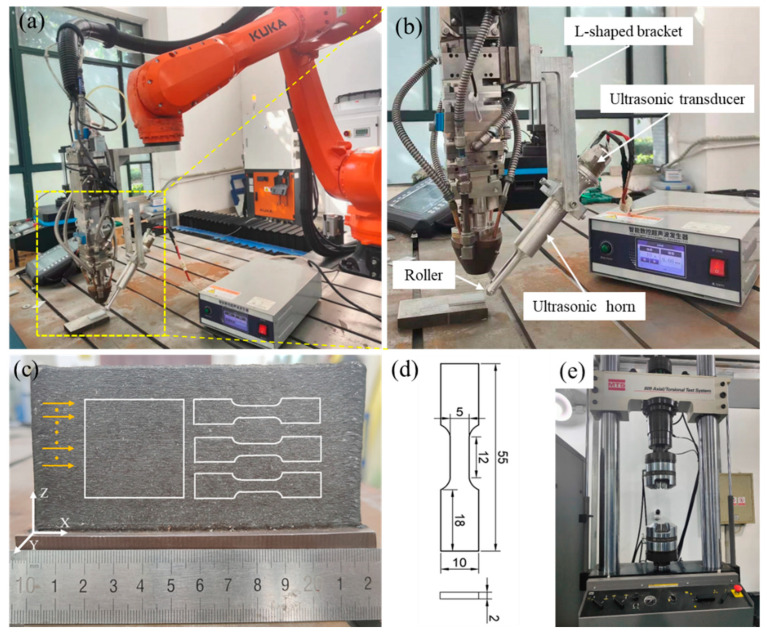
(**a**) LDED-UR device, (**b**) detailed structure of the ultrasonic rolling setup, (**c**) the manufactured thin-wall structure, (**d**) the size of the fatigue sample, and (**e**) fatigue testing machine.

To minimize pores and enhance the fatigue performance of alloys, an in situ ultrasonic rolling setup has been developed, as presented in Figure 2. An L-shaped bracket is designed to integrate the ultrasonic rolling device with the LDED system for synchronous manufacturing. Figure 2b shows the detailed structure of the ultrasonic rolling device. It can be found that the ultrasonic rolling equipment primarily consists of an ultrasonic transducer, a booster, and a roller. The larger portion of the booster is connected to the ultrasonic transducer, while the smaller portion is attached to the roller. The ultrasonic transducer converts ultrasonic signals into mechanical vibrations, which are then amplified and transmitted to the roller through the booster. The amplitude of the roller can be adjusted by modifying the ultrasonic power output of the generator. Additionally, the distance between the roller’s point of contact and the melt pool can be managed by adjusting the fixed height and rotation angle of the UR system. Meanwhile, the angle of inclination of the ultraviolent rolling head relative to the rolled surface is fixed at 45°.

The manufactured thin-wall structure is displayed in Figure 2c, along with the marked laser scanning strategy. Following several optimization tests, the optimized laser processing parameters were established as follows: a laser power of 1400 W, a scanning speed of 8 mm/s, a powder feeding rate of 8 g/min, a layer thickness of 0.3 mm, a laser spot diameter of 3 mm, and a shielding gas flow rate of 1 L/min. For the in situ ultrasonic rolling (UR) experiments, the chosen parameters include an ultrasonic power of 600 W, a frequency of 20 kHz, and a distance of 15 mm between the laser spot and the rolling point. The pressure force for the top for ultrasonic rolling on the surface of the sample is measured via a dynamometer, and the value is 200 N. Note that the details of the optimization experiments will not be discussed in this manuscript. A simple description is exhibited in the following. Firstly, the laser power and scanning speed are selected as the adjustable parameters. Secondly, laser power values of 1000 W, 1200 W, 1400 W, and 1600 W are selected. Meanwhile, scanning speed values of 6 mm/s, 8 mm/s, and 10 mm/s are used. The values of other parameters, such as the powder feeding rate, layer thickness, and laser spot diameter, are all set as fixed values, which are obtained from past experiences. Thirdly, the laser-directed energy deposition tests are carried out. Lastly, the optimized value of the LDED tests can be chosen by evaluating the forming quality and the fraction of pores. By comparing the forming surface quality of the 12 group tests, 5 groups are selected. Then, the selected groups are cut via the electric discharge machining (EDM) wire-cutting machine. By employing the optical microscope platform, the fraction of pores can be evaluated. The results show that the alloy presents the least fraction of pores under a laser power of 1400 W and a scanning speed of 8 mm/s. Therefore, the optimized parameters are obtained.

### 2.3. Microstructure Analysis

After deposition, the structures are machined by electric discharge machining (EDM) wire cutting into the geometry specified in Figure 2c. The left white square in Figure 2c is cut for microstructure observation using a Zeiss optical microscope (OM) platform. Prior to the microstructural observations, the samples were chemically etched for 20 s using a solution of 30 mL HCl and 10 mL HNO_3_ as the etchant. The etched surfaces are then cleaned with an ultrasonic cleaning setup to remove the residual etchant. An electron backscattered diffraction (EBSD) system is also used to characterize microstructures. Before the EBSD analysis, the samples were electrolytically etched for 15 s in a hybrid solution of 40 mL C_2_H_5_OH and 10 mL HClO_4_. After the EBSD operation, the OXFORD-HKL Channel 5.0 software is employed to analyze the data.

### 2.4. Microhardness Test

Microhardness tests were conducted to examine the impact of two distinct processes, LDED and LDED-UR, on the mechanical properties of the deposited alloy. A Vickers hardness tester (HVS-1000Z, Vegour, Shanghai, China) was utilized to measure the hardness of the YZ section. During the hardness tests, a load of 200 g was applied for a duration of 10 s. Measurements were taken at 1.5 mm intervals from the substrate to the uppermost layer of the deposition.

### 2.5. Fatigue Tests

In order to investigate the fatigue properties of the LDED and LDED-UR alloys, high-cycle axial tensile fatigue tests were carried out at room temperature following the GB/T 3075–2008 standard [39]. Sample dimensions are presented in Figure 2d, obtained from deposited structures (Figure 2c) through the electric discharge machining (EDM). Figure 2e illustrates the employed MTS809 fatigue testing machine (MTS, Eden Prairie, Minnesota, USA). Prior to testing, sample surfaces were carefully ground with varying grit sandpapers, and then polished using polycrystalline diamond and colloidal silica suspension to achieve a mirror finish. The cyclic loads applied were sinusoidal at a frequency of 30 Hz, with a stress ratio R of −1, where R = σ_min_/σ_max_. Fatigue life was defined by the number of cycles to failure, capped at 10^6^ cycles. If a specimen survived 10^6^ cycles without failure, loading ceased automatically. The fatigue limit in this study refers to the maximum stress corresponding to a fatigue life of 10^6^ cycles. To ensure data reliability, three specimens were tested per stress amplitude. Finally, scanning electron micrograph (SEM) analysis was employed to observe the fatigue fracture surfaces, providing insights into the fracture characteristics.

## 3. Results and Discussion

### 3.1. Microstructure Characterization

**Figure 3:**
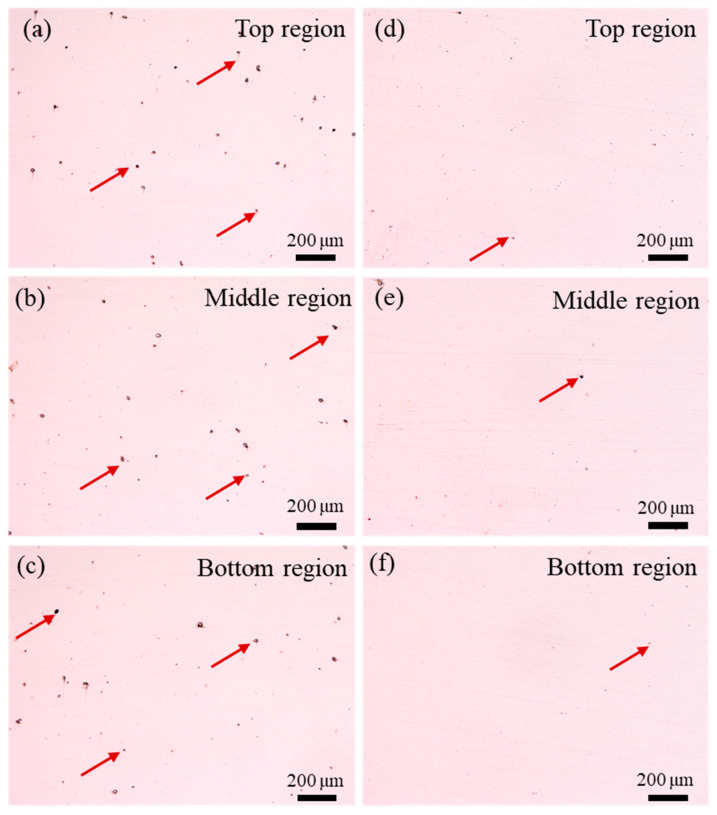
Pores of the LDED alloy (**a**–**c**) and LDED-UR alloy (**d**–**f**).

Figure 3 shows the porosity characteristics of the LDED and LDED-UR alloys across three representative regions. From Figure 3, it can be clearly seen that the fraction of pores is different for the two cases (LDED and LDED-UR). In the LDED alloy, a significant number of pores (marked by crimson arrows) are observed in Figure 3a–c, with an average size of about 34.1 μm. The appearance of these pores is primarily due to the instability of the melt pool [40,41]. When in situ ultrasonic rolling is applied to the LDED alloys, the pore fraction is dramatically decreased, as presented in Figure 3d–f. Meanwhile, the average size of pores decreases to about 10.2 μm after the application of in situ ultrasonic rolling. Archimedes density measurements were conducted to evaluate the density of the two alloys, and the results show that the density of the LDED and LDED-UR alloys is 98.26% and 99.27%, respectively. It can be summarized that both the fraction and size of pores are decreased in the LDED-UR alloy. This is attributed to the fact that the closure of pores is facilitated by the plastic deformation and metal flow induced by ultrasonic rolling.

**Figure 4:**
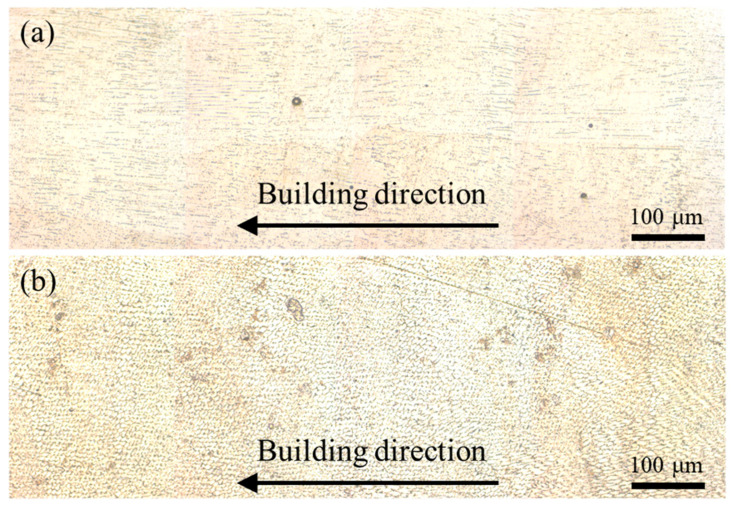
Optical micrograph of the LDED alloy (**a**) and LDED-UR alloy (**b**).

Figure 4 displays the grain morphology of the LDED and LDED-UR alloys along the building direction in the XZ section. From Figure 4a, it can be found that columnar dendrites form along the building direction when the alloy is deposited using LDED. The directional growth of grains along the [001] orientation during the laser-directed energy deposition process has been thoroughly discussed in our previous studies [42,43,44,45]. Meanwhile, the [001] orientation dendrites axes can be ascribed to the large temperature gradient and small crystal growth velocity. Note that the steep temperature gradients are mostly parallel to the building direction. In particular, the epitaxial growth of columnar microstructures is also correlated with the partially remelted grains of the previously deposited layer, which act as pre-nuclei for the directional growth of crystals. When the in situ ultrasonic rolling is applied, the fully equiaxed grains appear, as presented in Figure 4b. It is also indicated that the applied ultrasonic rolling effectively influences the entirety of the deposited layers.

**Figure 5:**
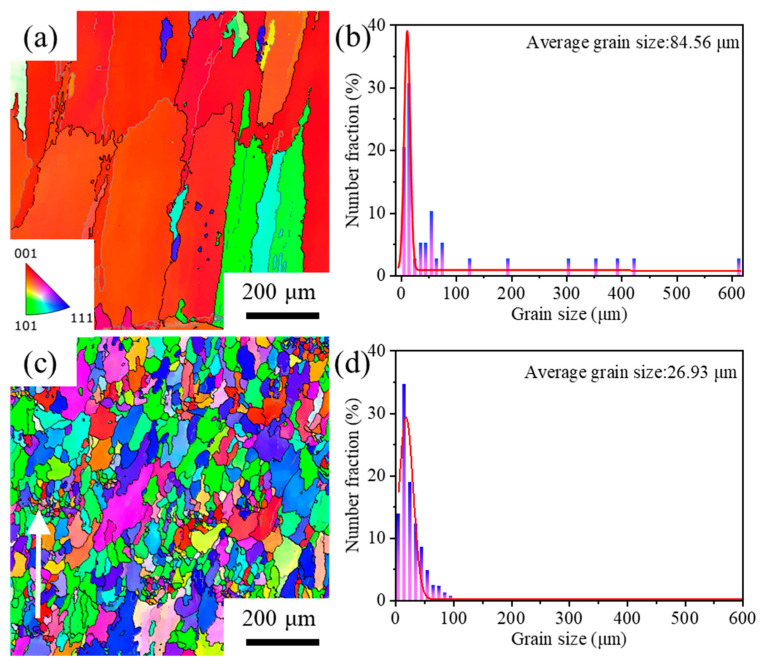
Inverse pole figure (**a**,**c**) and grain size distribution (**b**,**d**) of the LDED alloy (**a**,**b**) and LDED-UR alloy (**c**,**d**).

In order to further explore the grain characteristics of deposited alloys, EBSD (electron backscattered diffraction) analysis is employed. The middle regions of both the LDED alloy and the LDED-UR alloy are selected, and the results are presented in Figure 5. From Figure 5, it can be seen that distinct grain morphologies are observed in the LDED alloy and LDED-UR alloy, specifically the columnar dendritic grains and the equiaxed grains, respectively. This discovery aligns with the results illustrated in Figure 4. In Figure 5a, two types of grains are found: coarse grains and fine grains. The color of the coarse grains varies slightly. The fine grains are positioned between the coarse grains. The formation of fine grains is attributed to the recrystallization induced by the thermal stress [46]. By comparing the scale bar of the SEM image (200 μm) to the layer thickness (300 μm), it can be deduced that there are only a few deposited layers in the displayed figure. That is to say, the suggested ultrasonic rolling positively influences grain refinement. Additionally, from Figure 5c, it is noted that the grain size varies along the building direction (indicated by the white arrow). A gradient distribution of grains along the building direction is apparent. This suggests that grains can be effectively refined near the treated surface through the ultrasonic rolling process, with their impact gradually diminishing towards the substrate.

The average grain size is quantified to analyze the refinement effects of in situ ultrasonic rolling. For the LDED alloy, the average grain size is approximately 84.56 μm, as shown in Figure 5b. After in situ ultrasonic rolling, the average grain size of the deposited structure is significantly reduced to 26.93 μm (Figure 5d), which is substantially smaller than that of the LDED alloy. This quantitatively demonstrates that the application of in situ ultrasonic rolling on the LDED sample can significantly diminish the size of the grains.

**Figure 6:**
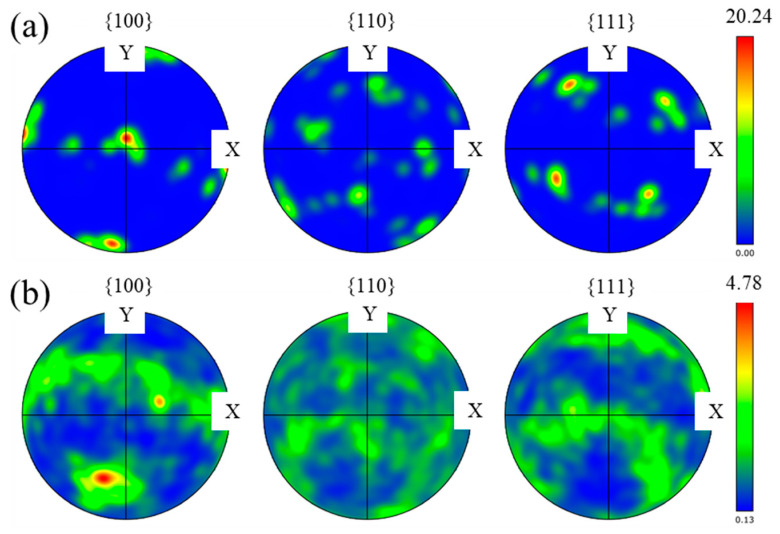
Pole figure of the LDED alloy (**a**) and LDED-UR alloy (**b**).

The pole figures are analyzed to discuss the microstructure anisotropy of two alloys. Figure 6 displays the pole figures in the {100}, {110}, and {111} orientations of two alloys. From Figure 6a, the maximum intensity of the pole figure is observed in the {100} orientation, with a value of 20.24. This is also can be found in the {100} orientation of the LDED-UR alloy (Figure 6b), where the maximum intensity is 4.78, significantly lower than that of the LDED alloy. The reduced maximum intensity of the pole figure for the LDED-UR alloys indicates that the microstructure anisotropy is substantially diminished in the LDED-UR alloy.

In summary, the grain size and microstructural anisotropy of the LDED-UR alloy are significantly reduced compared to those of the LDED alloy. The mechanisms behind these changes are discussed here. The appearance of equiaxed grains in the LDED-UR alloy can be attributed to two factors: ultrasonic vibration and plastic deformation induced by the roller. Ultrasonic vibration plays a primary role in grain refinement before the roller’s action. The fluidity of the melt pool and the semi-solid zone is enhanced by the combined effects of cavitation and acoustic streaming, which are induced by ultrasonic vibration. The enhanced fluidity allows bubbles to easily rise and escape from the melt pool, resulting in a notably decreased porosity fraction during the LDED-UR process. Concurrently, under the effects of cavitation and acoustic streaming phenomena, a large number of microscopic bubbles form in the melt pool and semi-solid zones. As the sound pressure attains a specific threshold, these minute bubbles expand rapidly and then collapse abruptly. This collapse generates substantial shockwave energy within the melt pool and the semi-solid region, causing the fragmentation and detachment of columnar grains. The resultant dendrites become new sites for the nucleation of equiaxed grains.

On the other hand, the roller’s substantial rolling pressure triggers severe plastic deformation, leading to dislocation generation. Concurrently, a high dislocation density arises from the combined impacts of the ultrasonic energy field, cyclic thermal field, and plastic deformation. Subsequently, these dislocations swiftly glide and coalesce within a brief period. This fusion of dislocations gives rise to small-angle grain boundaries, which transition into new grain boundaries. The process of dynamic recrystallization further refines these equiaxed grains.

### 3.2. Microhardness

**Figure 7:**
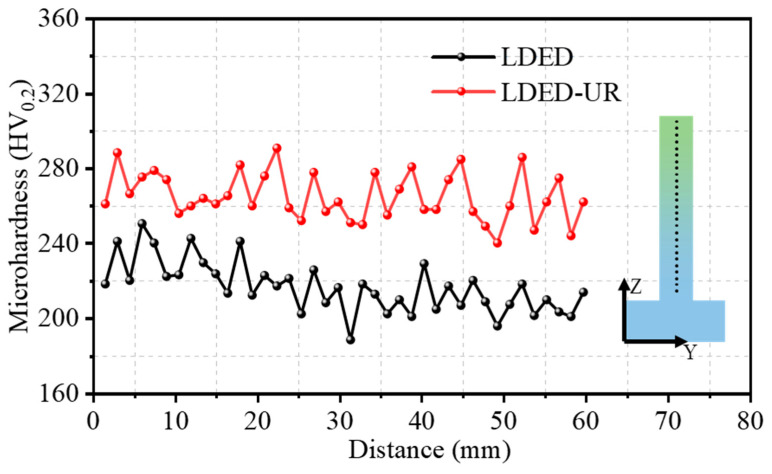
Microhardness of the LDED alloy and LDED-UR alloy.

The significantly distinct microstructure characterizations directly influence the mechanical properties of the LDED and LDED-UR alloys. In this paper, the mechanical properties of two samples are discussed in detail, and their underlying mechanisms are thoroughly investigated. Figure 7 presents the microhardness of the LDED and LDED-UR alloy along the building direction in the YZ plane. A total of 40 points were utilized to assess the microhardness of these two distinct samples. From Figure 7, it is evident that the microhardness variation along the deposition direction is minimal for both cases examined, suggesting a consistent distribution of microhardness throughout. The mean microhardness values for the LDED and LDED-UR samples are found to be 215.88 ± 11.83 HV_0.2_ and 264.78 ± 12.16 HV_0.2_, respectively. The enhanced microhardness in the LDED-UR sample can be attributed to the grain refinement induced by the in situ ultrasonic rolling process.

### 3.3. Fatigue Properties and Strengthening Mechanism

**Figure 8:**
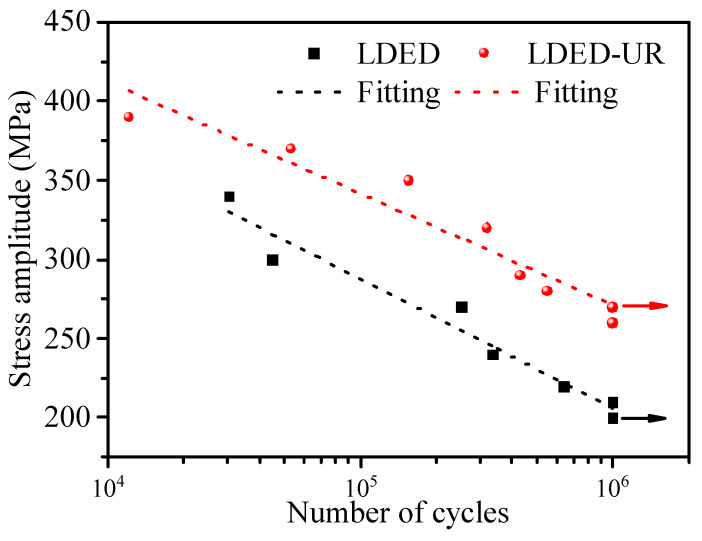
S-N curves of deposited 316L alloys manufactured by LDED and LDED-UR.

Figure 8 presents the results of the high-cycle fatigue (HCF) tests. Samples that withstood 10^6^ cycles without failure were deliberately halted, considering this number of cycles as their endurance limit. To enhance the visualization of the high-cycle fatigue behavior across different sample categories, data points from each category were subjected to linear regression analysis. The parameters of these regression lines are outlined in Table 1. From Figure 8, it is evident that the fatigue lives of the two alloys increase with the decrease in the stress amplitude. This opposite trend can be attributed to the reduction of maximum stress at a given stress ratio, which results in a reduction in dislocation nucleation [47]. As a result, the initiation of fatigue crack is delayed. Moreover, it can also be seen from Figure 8 that the fatigue limit (at the fatigue life of 10^6^ cycles) of the LDED alloy and the LDED-UR alloy are notably distinct. For the LDED alloy, the fatigue limit stands at 210 MPa. In contrast, for the LDED-UR alloy, the fatigue limit is considerably elevated to 270 MPa, marking an increase of 29% compared to the LDED alloy.

**Figure 9:**
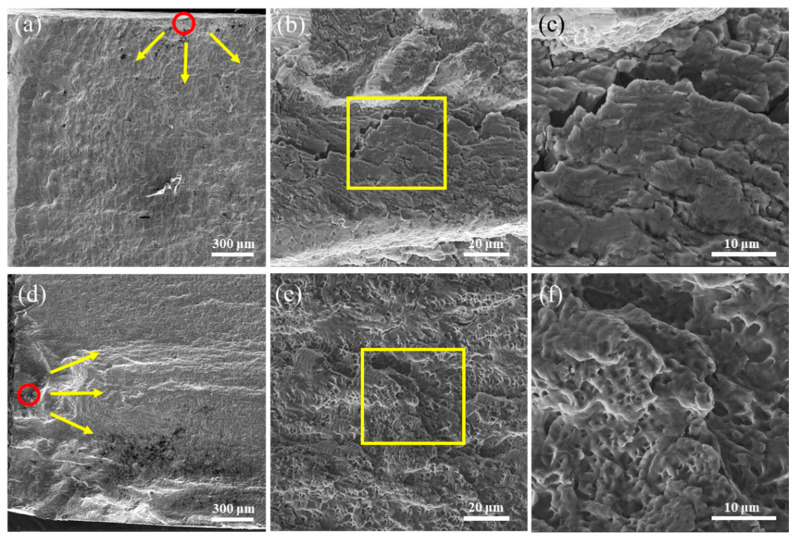
Macro- (**a**,**d**), meso- (**b**,**e**), and micrograph (**c**,**f**) of fatigue fracture morphologies of the LDED alloy (**a**–**c**) and LDED-UR alloy (**d**–**f**).

Figure 9 illustrates the fracture morphologies of the LDED alloy and LDED-UR alloy at different levels. The red circle in Figure 9a highlights the crack initiation site, while the yellow arrows show the direction of crack propagation. From Figure 9a, it is evident that the crack initiation site is located on the surface of the LDED alloy. In contrast, the LDED-UR alloy has a sub-surface crack initiation site, as illustrated in Figure 9d. This observation aligns with the findings reported in [48]. Meanwhile, this difference can be ascribed to the compression residual stress, which is introduced by surface cold working, i.e., in situ ultrasonic rolling. From Figure 9b,c and Figure 9e,f, it can be found that typical local fatigue striations appear in the fracture surfaces. For the LDED alloy, a pattern of flaky fracture can be discovered in the meso- and micrograph. Meanwhile, a pattern of flaky fracture (Figure 9e) transforming to the mixed brittle-ductile pattern (Figure 9f) can be found in the fatigue fracture morphologies of LDED-UR alloy. These results are consistent with the findings of Maruschak et al. [49]. It also can be concluded that the differences in the LDED and LDED-UR alloys are obviously at micro levels. The improved fatigue strength of the LDED-UR 316L stainless steel can be attributed to its low porosity and fine equiaxed grains. As illustrated in Figure 3, the fraction and size of pores in the LDED-UR alloy are significantly reduced via in situ ultrasonic rolling. Numerous studies [50,51] have highlighted that the fatigue strength is strongly correlated with the porosity characteristics of the additively manufactured alloy. Shrestha et al. [25] found that porosity serves as the precursor for fatigue crack initiation and significantly affects fatigue behavior. This phenomenon is primarily due to stress concentrations forming around micro-pores, which then leads to cracks forming under cyclic loading.

Aside from the porosity, the grain size also plays a pivotal role in determining the fatigue strength of the metal. This is evident from the optical micrograph (Figure 4) and the inverse pole figure (Figure 5), which clearly show that the grain size is dramatically decreased through in situ ultrasonic rolling. Our previous study [4] suggests that the fine grain addresses high tensile strength. The refined grain structure and increased strength of the LDED-UR alloy indicate that the dislocation motion and slip are effectively hindered, thereby inhibiting crack initiation [52]. Therefore, it can be concluded that in situ ultrasonic rolling is an effective method to enhance the fatigue strength of the LDED alloy.

## 4. Conclusions

A novel in situ ultrasonic rolling device has been developed and incorporated into the LDED operation, enabling each deposited layer to be shaped simultaneously. This study thoroughly explores the microstructural attributes and fatigue performance of alloys fabricated via the LDED and LDED-UR techniques. The primary conclusions are outlined below.

(1) The average size of pores in the LDED alloy is about 34.1 μm, which is larger than that of the LDED-UR alloy (10.2 μm). Meanwhile, the density of the LDED-UR alloy is improved from 98.26% (LDED alloy) to 99.27%.
(2) The average grain size of the LDED-UR alloy is 26.93 μm, which is far smaller than that of the LDED alloy (84.56 μm). The fully equiaxed grains are obtained by in situ ultrasonic rolling technique, and then the microstructure anisotropy is greatly reduced.
(3) The fatigue limit of the LDED-UR alloy is 270 MPa, which is 29% higher than that of the LDED alloy (210 MPa). For the LDED-UR alloy, it has a sub-surface crack initiation site, which is different from that of the LDED alloy.
(4) The enhanced fatigue limit of the LDED-UR alloy is strongly correlated with the reduced porosity and the fine grains.

## Figures and Tables

**Table 1:** Parameters of the trend lines for the fatigue data and the corresponding R^2^.

Samples	Fitting Parameters	R^2^
LDED	−81.54N + 695.32	0.9589
LDED-UR	−70.51N + 694.14	0.9241

## Data Availability

The raw data supporting the conclusions of this article will be made available by the authors on request.
